# Presence of Atrial Fibrillation in Stroke Patients With Patent Foramen Ovale: Systematic Review and Meta-Analysis

**DOI:** 10.3389/fneur.2021.613758

**Published:** 2021-04-15

**Authors:** Jessie Ze-Jun Chen, Vincent N. Thijs

**Affiliations:** ^1^Department of Neurology, Austin Health, Heidelberg, VIC, Australia; ^2^Florey Institute of Neuroscience and Mental Health, University of Melbourne, Melbourne, VIC, Australia

**Keywords:** atrial fibrillation, patent foramen ovale, ischaemic stroke, transient ischaemic attack, cryptogenic stroke

## Abstract

**Purpose:** Patent foramen ovale (PFO) is associated with ischemic stroke, especially in patients with embolic stroke of undetermined source. This study aims to evaluate the presence of atrial fibrillation (AF) in ischemic stroke patients with PFO.

**Methods:** We systematically searched EMBASE and MEDLINE databases on May 21, 2020 for studies that analyzed the presence of AF in patients with PFO. The primary outcome was the presence of AF in patients with PFO compared with those without. Outcomes were pooled using a random-effects model using the method of DerSimonian and Laird. We recorded demographic characteristics and the methods used for AF detection in the studies included (unspecified, history/medical records review, ECG, Holter monitor, or loop recorder).

**Results:** A total of 14 studies and 13,245 patients fulfilled the entry criteria. The average age was 61.2 years and 41.3% of the participants were female. There was a lower risk of AF in patients with PFO compared with those without (RR 0.52, 95% confidence interval, 0.41–0.63, *p* < 0.001). There was no evidence of heterogeneity. The lower risk of AF was found in cross-sectional and longitudinal studies and in studies stratified by average age (<60 or ≥60) and in cryptogenic stroke. Meta-regression by PFO detection technique suggested that studies using transoesophageal echocardiogram for PFO detection reported higher risk of AF (1.39, 95% confidence interval 1.14–1.70, *p* = 0.004).

**Conclusion:** The presence of a PFO in patients with ischemic stroke/TIA may be associated with a lower risk of AF. Few studies have estimated the risk of future AF in patients with PFO.

## Introduction

A patent foramen ovale (PFO) is present in 20–25% of the general population and in up to 50% of younger stroke patients (1). Case-control studies have shown that PFO is strongly associated with ischemic stroke, especially in younger patients and in cryptogenic stroke (1–6). This has led to randomized controlled trials (7–11) that showed that percutaneous PFO closure reduces future stroke risk.

Before considering closure of PFO, the cryptogenic nature of the stroke needs to be demonstrated. Atrial fibrillation (AF) is an established risk factor for stroke. Although AF generally occurs in the elderly, it can occur in the age range where a PFO is considered an etiologic factor. Ruling out paroxysmal AF as an etiologic factor in this population is difficult.

The aim of this study was to perform a systematic review and meta-analysis of the available literature in order to determine the risk of AF in patients with ischemic stroke who have PFO as compared to those without PFO. Clinical variables that are associated with AF detection will be explored, as this may inform the selection of PFO patients for prolonged cardiac monitoring. AF associated with PFO closure is outside the scope of this review.

## Methods and Materials

This systematic review and meta-analysis was registered with PROSPERO (The International Prospective Register of Systematic Reviews; CRD42019109505) and follows the PRISMA guideline for meta-analysis reporting.

### Search Strategy

Articles for review were retrieved by searching the databases MEDLINE and EMBASE (inception to 21st May, 2020), using the key terms “patent foramen ovale,” “atrial septal defect,” “atrial fibrillation,” “atrial flutter,” “atrial arrhythmias,” “closure,” “transcatheter closure,” “surgical closure,” “ischemic stroke,” “cryptogenic stroke,” and associated MeSH headings (Supplementary Table 1). The title and abstract screen were performed independently by V. T. and J. C., using the Rayyan tool (12). Full text review of the remaining articles was performed by J. C. This strategy was supplemented by a manual search of reference lists from key articles.

### Inclusion and Exclusion Criteria

We considered all original research, including prospective or retrospective cohort studies, case series, and comparative studies. We included cross-sectional studies that reported on the co-detection rate of PFO and AF, and studies that evaluated the rate of AF on longitudinal follow-up of ischemic stroke patients with and without PFO. Studies of AF post PFO closure were not included in this review. Studies with fewer than 50 patients were excluded. We excluded composite studies that examined both PFO and atrial septal defect closure, unless PFO-specific outcomes were separately reported and the PFO component contained 50 or more patients. We also excluded abstracts and studies in non-stroke populations. Publications were evaluated for duplicate or overlapping data, and only the most complete studies were included. Unpublished data were not sought.

### Quality and Bias Assessment

Assessment for study quality and bias was performed by J. C. and V. T. using the SIGN tool (13). Conflict was resolved by discussion and consensus.

### Data Extraction

Data extraction was performed by J. C. using a standardized Excel worksheet. We collected information on the principal author, year of publication, study design, sample size, methods for PFO detection—transcranial Doppler (TCD), transthoracic echocardiogram (TTE), transesophageal echocardiogram (TOE), and methods for AF detection (unspecified, medical records, history/questionnaire, electrocardiogram (ECG), Holter monitor of at least 24 h duration, or loop recorder). We also collected clinical variables known to predispose patients to AF, including average age, proportion of females, and proportion of patients with hypertension and diabetes.

### Statistical Analysis

For all analyses, we adopted a random effects model using the Der Simonian-Laird method. Additionally, we used the Sidik-Jonkmann method for sensitivity analysis (14). These methods assume that different studies are estimating different but related effect sizes and are a more conservative approach compared to fixed effects model when heterogeneity is present.

For the risk of AF in patients with PFO compared to those without, we performed meta-analysis of proportions using the Stata (ver 15.1, StataCorp, College Station, Texas) *metan* command. The pooled estimates were expressed as relative risk. All pooled estimates were presented with their 95% confidence intervals and 2-tailed *p*-values. A *p* < 0.05 was considered statistically significant.

Heterogeneity of the results was tested using the chi square, *I* squared (15) and Tau-squared tests. Heterogeneity was considered low if *I*^2^ <25%, moderate if *I*^2^ is between 25 and 50%, and significant if *I*^2^ > 50% (15). A *p* < 0.10 was considered statistically significant due to the lower power of these tests in meta-analyses where studies have smaller sample sizes or are few in number. Meta-regression was performed to assess the contribution of each pre-specified variable (i.e., age, proportion of females, proportion of patients with hypertension and diabetes, and methods of AF and PFO detection) to the overall risk of AF.

Publication bias was assessed graphically using the funnel plot and further assessed using Egger's regression asymmetry testing (16). The intercept of the linear regression line with the *y*-axis is used to measure asymmetry. If the intercept is significantly different from zero, this suggests the presence of publication bias.

## Results

### Study Selection

**Figure 1:**
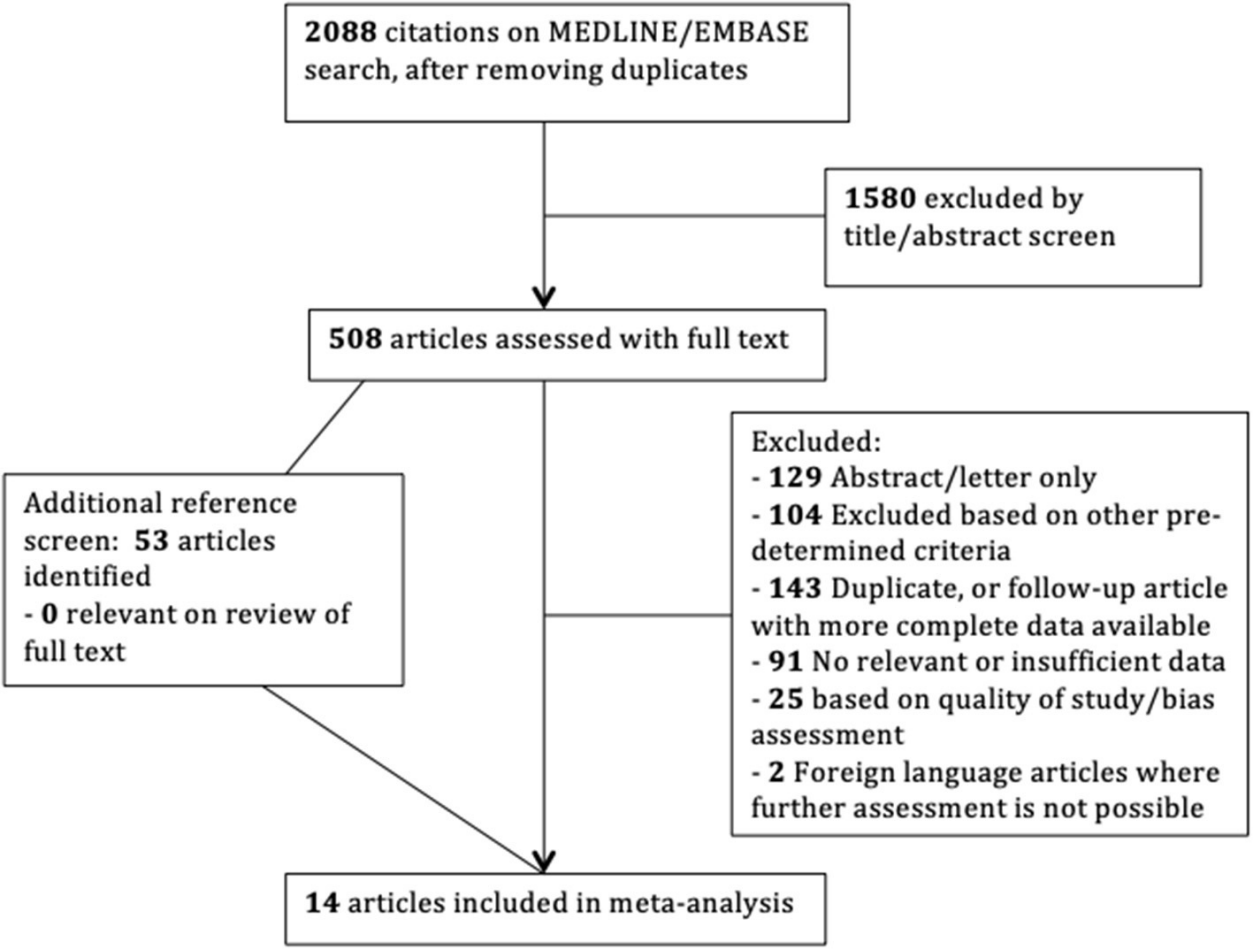
Study selection flowchart.

The search strategy retrieved 2,088 abstracts for review, and 1,580 of these were considered inappropriate following title/abstract screen. The remaining 508 articles were reviewed in full. References of included articles were screened by J. C., and no additional study was identified for inclusion in the final analysis. The progress through each step of the review process resulted in a final number of 14 studies included (Figure 1).

### Bias Analysis

Bias analysis for studies included is summarized in Supplementary Table 2. Overall, studies minimized selection bias by including consecutive patients from the ischemic stroke population. Four studies (17–20) included consecutive stroke patients who were referred for echocardiogram. This could be a source of selection bias, as this population may be different from the unselected ischemic stroke population. However, while the indication for echocardiogram referral was not explicitly stated, both AF and non-AF patients were included in these studies. Attrition bias could not be assessed in some studies, as the completeness of follow-up was not reported. Detection bias was an issue for some studies, due to the use of only chart review or ECG to detect AF. This likely leads to significant under-detection of AF. Lastly, two studies (19, 21) suffer from confounding bias, as important AF risk factors such as hypertension were not reported.

### Risk of AF in Patients With PFO Compared With Those Without PFO

**Figure 2:**
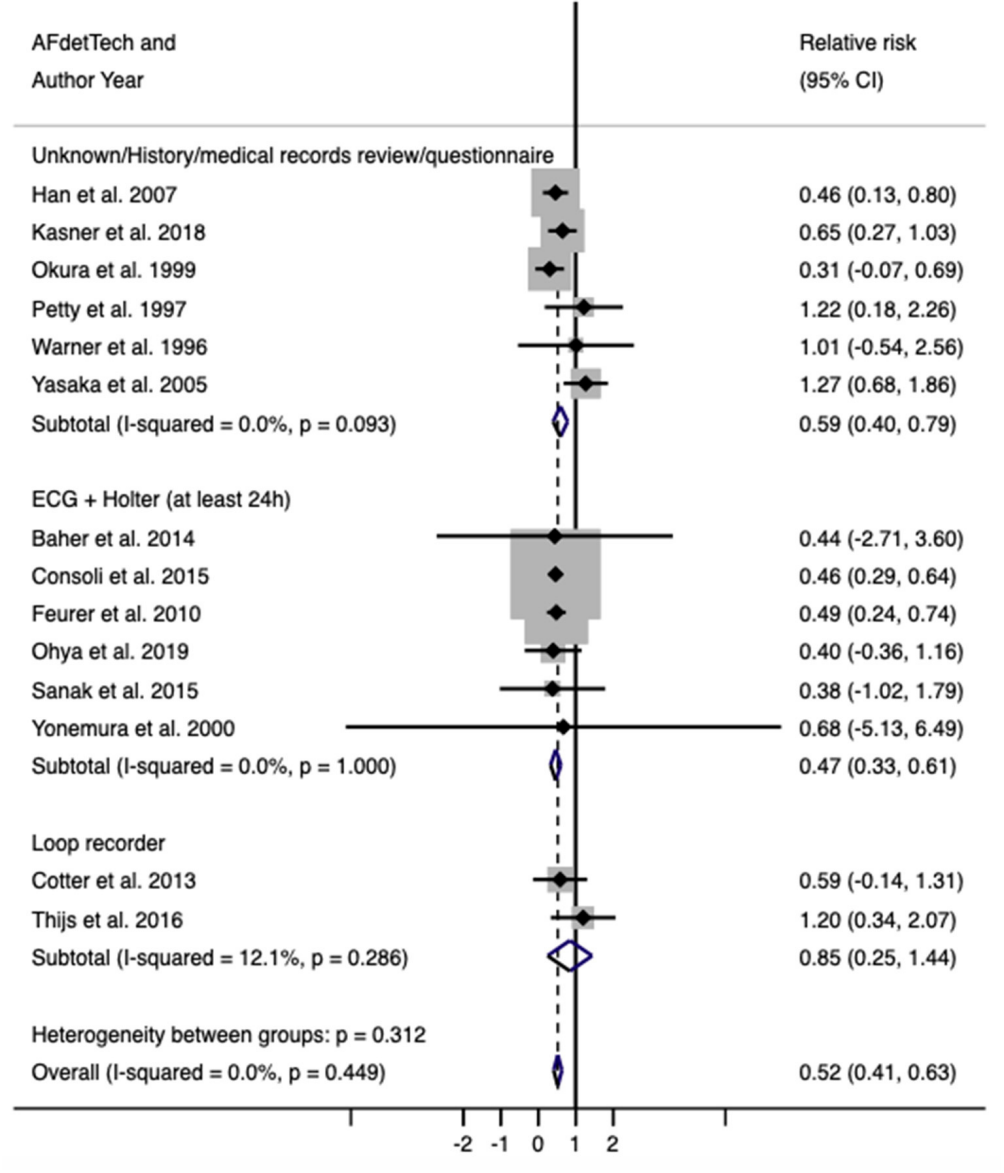
Relative risk (with 95% confidence interval) of atrial fibrillation in patients with PFO compared with those without.

A total of 14 studies (17–30) and 13,245 patients were included in this part of the analysis. Six of these studies (21–23, 25, 26, 30) reported on the frequency of AF, frequency of PFO, and frequency of AF and PFO co-detection in an unselected ischemic stroke population that underwent a standard stroke etiology work-up, including a 24 h Holter. Four of these studies (17–20) reported on the same results but included only stroke patients referred for echocardiogram. One study (29) included patients in whom the initial in-hospital investigations, including continuous ECG monitoring, were unrevealing. Two studies (24, 27) reported patients who underwent more prolonged AF monitoring after initial negative investigations and reported on the risk of AF in those with PFO compared with those without. Full characteristics of each study are detailed in Table 1. The average age was 61.2 years, and 42.1% of the participants were female. There was a reduced risk of AF detection in patients with PFO compared to those without (RR 0.52, 95% confidence interval, 0.41–0.63, *p* < 0.001; Figure 2). There was no evidence of heterogeneity (*I*^2^ = 0%, *p* < 0.001). Sensitivity analysis using the Sidik–Jonkmann method yielded identical results.

**Table 1:** Study characteristics—risk of AF in those with PFO compared to those without.

**References**	**Study type**	**Sample size**	**Average age**	**Female (*n*)**	**PFO detection**	**AF detection**	**Mean duration of AF monitoring**	**HTN (*n*)**	**Diabetes (*n*)**	**Non-index stroke/TIA (*n*)**
Baher et al. (22)	Prospective cohort	85	66	45	2	3	N/A	68	24	20
Consoli et al. (23)	Prospective cohort	1,130	68	453	1	3	N/A	793	234	N/A
Cotter et al. (24)	Prospective cohort	51	52	23	2	4	Median duration prior to first episode of AF= 48 days. Mean duration in those without AF= 229 days.	N/A	N/A	N/A
Feurer et al. (25)	Retrospective cohort	763	58	314	1	3	Minimum 24 hours	462	126	N/A
Han et al. (21)	Retrospective cohort	2,482	63	964	0	2	N/A	N/A	N/A	N/A
Okura et al. (17)	Prospective cohort	77	77	30	3	0	N/A	41	16	N/A
Petty et al. (18)	Retrospective cohort	116	63	NA	3	1	N/A	61	22	44
Šanák et al. (26)	Prospective cohort	98	40	42	3	3	3 weeks and 1 day.	5	1	NA
Warner et al. (19)	Retrospective cohort	106	66	51	3	0	N/A	N/A	N/A	N/A
Yasaka et al. (20)	Retrospective cohort	426	N/A	N/A	3	0	N/A	N/A	N/A	N/A
Thijs et al. (27)	Prospective cohort, *post-hoc* analysis	221	62	79	3	3	3.69 years (total 815.5 patient-years)	144	34	59
Kasner et al. (28)	RCT, *post-hoc* analysis	7,209	67	2,777	2	0	N/A	5,581	1,805	1,258
Ohya et al. (29)	Retrospective cohort	348	72	148	3	3	Minimum 24 h	271	103	68
Yonemura et al. (30)	Retrospective cohort	133	43	41	2	3	24 h	54	19	N/A

*Categories for AF detection methods*.

*0 = Not systematically approached or unknown; 1 = History/medical records review/questionnaire; 2 = ECG ± symptom driven Ix (ECG/Holter); 3 = ECG + Holter (at least 24h); 4 = Loop recorder*.

*Categories for PFO detection methods*.

*0 = Not 100% patients had assessment; 1 = TCD ± TTE; 2 = TTE ± TOE; 3 = TOE*.

*HTN, hypertension; DM, diabetes mellitus; RCT, randomized control trial; N/A, not available*.

Subgroup analysis based on AF detection techniques showed that the reduced risk of AF in PFO patients is seen across all subgroups. However, the effect estimate for the loop recorder subgroup has a wide confidence interval (RR = 0.83, 95% CI = 0.24–1.42), likely attributed to the small number of included studies. Four studies (24, 27–29), corresponding to 7,829 patients, specifically reported data on cryptogenic stroke. Subgroup analysis on these studies again showed reduced risk of AF in PFO patients (RR = 0.67, 95% CI = 0.38–0.96), as did studies that included all stroke subtypes (RR = 0.51, 95% CI = 0.37–0.65).

Univariable random-effects meta-regression by mean patient age, proportions of hypertension and diabetes, and method of AF detection did not detect an association with the risk of AF (Supplementary Table 3). Meta-regression by PFO detection technique suggested that studies using TOE for PFO detection reported higher risk of AF (1.39, 95% CI 1.14–1.70, *p* = 0.004).

**Figure 3:**
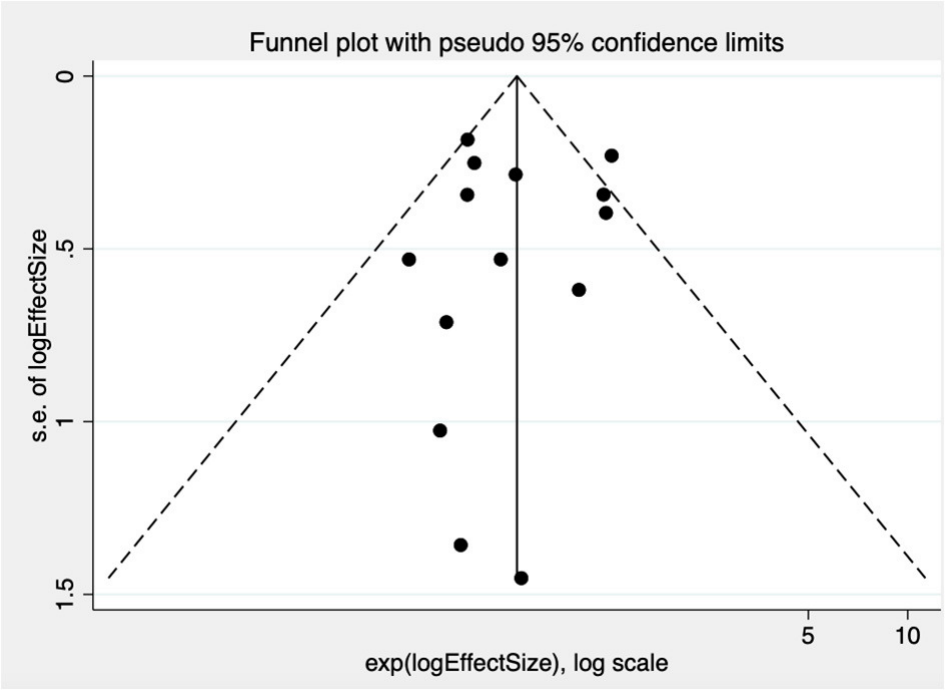
Funnel plot test for publication bias in studies examining risk of AF in patients with PFO compared with those without. Treatment effect is on the *x*-axis, measure of study size on the *y*-axis. s.e. indicates standard error.

There is no statistical evidence of publication bias (intercept = −0.09, *p* = 0.905), although the funnel plot suggested an absence of small studies reporting a higher risk of AF in those with PFO (Figure 3).

## Discussion

This study-level meta-analysis found that the presence of PFO is associated with a lower risk of AF detection in patients with ischemic stroke/TIA.

The lower risk of AF in patients with PFO compared with those without is consistent with the general view that patients with PFO are not at an increased risk of arrhythmias compared with the general population (31). In addition, studies have also demonstrated that the presence of AF reduces the likelihood of right-to-left shunting through the PFO due to the elevation of left atrial pressure and the change in the pressure gradient across the PFO (32, 33). This in turn reduces the likelihood of PFO and AF co-detection. Studies that used TOE for PFO detection reported a higher risk of AF, although the magnitude of this effect was small. Whether use of TOE is a proxy for performing more thorough assessment and prolonged monitoring for AF is unknown. While age was not found to be a significant contributor to heterogeneity in this analysis, there was not a high degree of variability in mean age across studies. The relationship between age and risk of AF may be different within studies. For example, the study by Yasaka et al. (20) which included patients of all ages, found that risk of AF was higher in PFO patients who were older. Lastly, a history of previous or recurrent cerebrovascular events may have been an important clinical factor that helps stratify the risk of AF. However, these data were reported in only three of the studies included and could not be examined adequately.

Our findings may have possible implications for diagnostic screening pathways after stroke and TIA. Knowing that a PFO is present, especially in a younger patient, may help determine the intensity of the monitoring regime for AF and avoid very prolonged monitoring. This may be particularly important in resource-limited settings.

There are several limitations to this study. First, two of the studies included (18, 21) suffered from detection bias (Supplementary Table 2), as they utilized routine ECGs, with or without once-off or symptom-triggered 24-h Holter monitoring for baseline and follow-up AF detection. Four studies (17, 19, 20, 28) did not explicitly state their method of AF monitoring. It is known that AF is often paroxysmal and asymptomatic, and these methods likely lead to under-detection of AF. This was illustrated by the CRYSTAL AF trial (34), which reported a much higher rate of AF of 12.4% at 12 months with insertable cardiac monitors. This is in contrast to the rate of 2% in the control group, where a mix of ECG and Holter monitoring were performed at the discretion of the clinician. The 2016 European Society of Cardiology Guidelines for the Management of Atrial Fibrillation (35) recommends that at least 72 h of continuous cardiac monitoring be performed for patients with ischemic stroke/TIA. In the 2014 American Heart Association/American Stroke Association guidelines, prolonged rhythm monitoring for 30 days is considered reasonable for patients who have had an ischemic stroke/TIA with no apparent cause (36). In the absence of adequate AF monitoring, the true incidence of AF may be higher.

Second, the methods for diagnosing PFO were heterogeneous, and there may be some detection bias if TTE is used as the sole modality to rule out a PFO. Third, all but four studies (24, 27–29) have reported data on an unselected ischemic stroke population. The inclusion of patients with a non-embolic stroke (such as a lacunar stroke), for whom a PFO is not considered a potential etiologic factor, may reduce the generalizability of the results. Furthermore, this is a study-level meta-analysis, and the relationships described are observational associations across trials and are prone to bias from unmeasured confounders. Adjusted summary statistics were available only for two studies (24, 27) and were included in all analyses. Examination of individual patient data will help to confirm these associations and offer valuable opportunities to study the impact of other important variables, such as PFO morphology, on the rate of AF. Lastly, there is a degree of publication bias resulting from the lack of small studies reporting a higher risk of AF in those with PFO.

## Conclusion

Stroke patients with PFO have a lower risk of AF compared with those without. Future research in this area should ensure adequate evaluation for AF over longer periods of cardiac monitoring and utilize a more rigorous AF follow-up protocol to determine the true incidence of AF.

## Data Availability Statement

The original contributions presented in the study are included in the article/Supplementary Material. Further inquiries can be directed to the corresponding author/s.

## Author Contributions

JC contributed to data collection, data analysis and interpretation, and drafted the manuscript. VT conceived and designed the analysis, contributed to data analysis, and critically revised the manuscript. All authors contributed to the article and approved the submitted version.

## Conflict of Interest

VT serves on the advisory board and receives consulting and speaker fees from Medtronic, Pfizer/BMS, and Bayer and Boehringer Ingelheim. The remaining author declares that the research was conducted in the absence of any commercial or financial relationships that could be construed as a potential conflict of interest.
