# Supplementary material for: Presence of Atrial Fibrillation in Stroke Patients With Patent Foramen Ovale: Systematic Review and Meta-Analysis
Source: Front Neurol. 2021 Apr 15;12:613758. doi: 10.3389/fneur.2021.613758 (PMC8081982; doi:10.3389/fneur.2021.613758)
Supplement: Supplementary file 1 [file Data_Sheet_1.docx]

**Supplementary Material**

**Presence of Atrial Fibrillation in stroke patients with Patent Foramen Ovale:**

**Systematic Review and Meta-analysis**

**Table I Search Strategy**

The search strategy used for Ovid MEDLINE (inception – 21 May 2020) was:

1. Atrial Fibrillation/

2. ((atrial or atrium or auricular) adj2 (fibrillat* or flutter* or tachycardia* or tachyarrhythmia*)).tw.

3. Atrial Flutter/

4. Tachycardia, Ectopic Atrial/

5. 1 or 2 or 3 or 4

6. Heart Septal Defects, Atrial/ or Foramen Ovale, Patent/

7. (patent foramen ovale or PFO).tw.

8. ((atrial or atrium) adj3 sept* adj3 defect*).tw.

9. ((right to left or venous to arterial or venous arterial) adj3 shunt).tw.

10. 6 or 7 or 8 or 9

11. 5 and 10

12. exp Stroke/ or ((cerebral or cerebro*) adj isch*mi*).mp. or ((cerebral or cerebro*) and (thromb* or embol*)).mp. or (brain adj (infarct* or lesion*)).mp. or (stroke* or cerebrovascular accident* or cerebral infarct*).mp. or cryptogenic stroke.mp.

13. (transcatheter closure or endovascular closure or device closure or percutaneous closure or PFO closure).mp.

14. 12 or 13

15. 11 and 14

Mapping to EMBASE was performed by expert librarian and duplicate results removed to yield final article count of 2088.

**Table II- Bias assessment of included studies**

| **Author** | **Year** | **Selection Bias** | **Attrition bias** | **Detection bias** | **Confounding** |
| --- | --- | --- | --- | --- | --- |
| Baher et al. | 2014 | No | No | No | No |
| Consoli et al. | 2015 | No | No | No | No |
| Cotter et al. | 2013 | No | Can't say | Yes | No |
| Feurer et al. | 2010 | No | Yes | No | No |
| Han et al. | 2007 | No | No | Yes | Yes |
| Okura et al. | 1999 | Yes | N/A | Can't say | No |
| Petty et al. | 1997 | Yes | N/A | Yes | No |
| Sanak et al. | 2015 | No | No | No | No |
| Warner et al. | 1996 | Yes | N/A | Can't say | Yes |
| Yasaka et al. | 2005 | Yes | N/A | Yes | No |
| Thijs et al. | 2016 | No | Can't say | No | No |
| Kasner et al. | 2018 | No | Can't say | Can't say | No |
| Ohya et al. | 2019 | Yes | No | Yes | No |
| Yonemura et al. | 2000 | No | No | Yes | No |

N/A: not applicable

**Table III- Random effects meta-regression results**

| **Variable** | **No. of studies** | **Co-efficient** | **Lower 95% CI** | **Higher 95% CI** | ***p* value** |
| --- | --- | --- | --- | --- | --- |
| Age | 13 | 0.99 | 0.94 | 1.03 | 0.52 |
| Female, % | 12 | 0.04 | 5.24e-6 | 296.93 | 0.44 |
| Hypertension, % | 10 | 0.99 | 0.05 | 20.55 | 1.00 |
| Diabetes, % | 10 | 0.18 | 0.00 | 523.91 | 0.64 |
| Non-index stroke/TIA, % | 5 | 40.16 | 0.02 | 65395.04 | 0.21 |
| PFO detection technique | 14 | 1.39 | 1.14 | 1.70 | 0.004 |
| AF detection technique | 14 | 0.92 | 0.75 | 1.14 | 0.42 |
